# Chemical Analysis of the Resinous Exudate Isolated from *Heliotropium taltalense* and Evaluation of the Antioxidant Activity of the Phenolics Components and the Resin in Homogeneous and Heterogeneous Systems

**DOI:** 10.3390/molecules14061980

**Published:** 2009-06-02

**Authors:** Brenda Modak, Macarena Rojas, René Torres

**Affiliations:** Departamento de Ciencias del Ambiente, Facultad de Química y Biología, Universidad de Santiago de Chile, Santiago, Chile; E-mail: maquitarojas@yahoo.com (M.R.), rene.torres@usach.cl (R.T.)

**Keywords:** flavonoids, antioxidant activity, *Heliotropium taltalense*, DPPH, SDS, micelles

## Abstract

*H. taltalense* (Phil.) Johnst. (Heliotropiaceae) is an endemic species of the northern coast of Chile that produces a resinous exudate that covers its foliar surface and stems. Its chemical composition was analyzed for the first time, and two aromatic geranyl derivatives: filifolinol and filifolinyl senecionate and three flavonoids – naringenin, 3-O-methylgalangin and 7-O-methyleriodictiol – were isolated. The antioxidant activity of the flavonoids and the resinous exudates was carried out by measuring the 1,1-diphenyl-2-picrylhydrazyl (DPPH) bleaching effect in ethanolic solution and in sodium dodecyl sulfate (SDS) micelles. The influence of the reaction medium was analyzed. The initial velocity reactions for the pure compounds and for the extract were higher in SDS media than in ethanolic solution. The velocity of reaction observed was interpreted in terms of the reaction medium environment in the micelle.

## 1. Introduction

*Heliotropium taltalense* (Phil.) Johnst., family *Heliotropiaceae*, *Cochranea* section, is an endemic resinous bush that grows in the north of Chile. *Heliotropium* species grow in arid regions with extreme environmental conditions and produce a resinous exudate from the trichomes that covers its foliar surface and stems. Phytochemical research revealed that this exudate is constituted by a mixture of different compounds, mainly flavonoids and aromatic geranyl derivatives [1,2,3,4,5,6,7,8]. The predominant occurrence of resinous exudates in plants growing in arid and semi-arid areas has been explained as a defense mechanism in terms of the extreme environmental conditions [9]. The indumentum of non-glandular trichomes and the lipophilic substances secreted by glandular trichomes (terpenoids, lipids, waxes and flavonoid aglycones) serve as a barrier against diverses external factors, including herbivores and pathogens, UV-B radiation, extreme temperatures and excessive water loss [10]. It has also been proposed that the presence of antioxidants in the exudates, as flavonoids, can prevent the oxidative degradation of other of its constituents as the terpenoids [11]. Some of the possible consequences of this interaction are a direct modulation of membrane physical properties on the capacity of flavonoids to efficiently scavenge free radicals, in part inhibiting the lipid oxidation chain reaction. The more hydrophilic flavonoids interact by hydrogen bonding with the polar head groups at the lipid–water interface of membranes. This type of interaction may provide a level of protection for the bilayer from external and internal aggressors (i.e. oxidants) contributing to preserve the structure and function of biological membranes [12].

The structures of the flavonoids are adapted to capture free radicals due to the easiness with which the hydrogen of the hydroxyl group can be donated to the radical and to generate a more stable structure [13]. Therefore, the number and localization of the hydroxyl groups would be clearly involved in the antioxidant activity of these compounds [14,15,16].

The flavonoids are important components of the diet and several research studies have reported their beneficial antioxidant properties for health. However, the antioxidant activity of this type of constituents of vegetable resinous exudates, where the complexity of the matrix and the presence of high solar ultraviolet radiation in the environment can make of them an important defense mechanism, has not been well studied. In this regard, studies consisting in their antioxidant evaluation, have mostly been carried out in homogeneous systems, without considering their hydrophobic properties. A study employing the lipophilic antioxidant α-tocopherol, β-carotene and ubiquinol showed their incorporation into membranes and lipoproteins. They can be localized at the surface or interior. The long side chains of tocopherols, tocotrienols, and ubiquinols which are essential for their incorporation and detainment in the membranes and lipoproteins, reduce the mobility within and between both. Therefore, the antioxidant activity may vary dramatically in the membranes and lipoproteins. Furthermore, localization and mobility at the microenvironment are important, especially for lipophilic antioxidants [17]. 

A method that considers this property is the use of microheterogeneus systems, such as micelles. They can be pictured as having a non-polar interior and relatively polar interfacial region [18]. Micelles of ionic surfactants can interact electrostatically with highly polar solutes because the large surface charge densities of these aggregates lead to strong ion-dipole interactions [19]. Thus, uric acid, a water soluble antioxidant, is able to react with micelle incorporated DPPH radicals (liposoluble radical) in the interface lipid/water of micelles. This result can be explained in terms of the compartmentalization of the reactants in different environments and the effect of electrostatic interactions in modulating the access of the urate anion to the micelar interface [20].

On the other hand, the total reactive antioxidant of resinous exudates and flavonoids of other *Heliotropium* species were previously evaluated by measuring the bleaching of stable free radicals (ABTS and DPPH) in ethanol solution [21]. In that research, all flavonoids tested reacted at intermediate rates. A poor correlation between the charges of antioxidants evaluated by the ABTS and DPPH procedures was also observed. This poor correlation can be attributed to the evaluation of total antioxidant charge in complex mixture that is extremely dependent upon the procedure employed, concluding that much care must be exercised in the interpretation of the results.

Based in these antecedents, we now report the results obtained on the evaluation of the antioxidant activity of the resinous exudate and the flavonoids isolated from *Heliotropium taltalense* Phil., using two different reaction media. Specifically, the antioxidant activity was carried out evaluating the absorbance changes of an ethanolic solution of DPPH (homogeneous system) and using a micelar solution of sodium dodecyl sulfate, an anionic surfactant (heterogeneous system). The phytochemical analysis of resinous exudates isolated from *Heliotropium taltalense* Phil. is also reported for the first time.

## 2. Results and Discussion

### 2.1. Chemical composition of the resin

**Figure 1:**
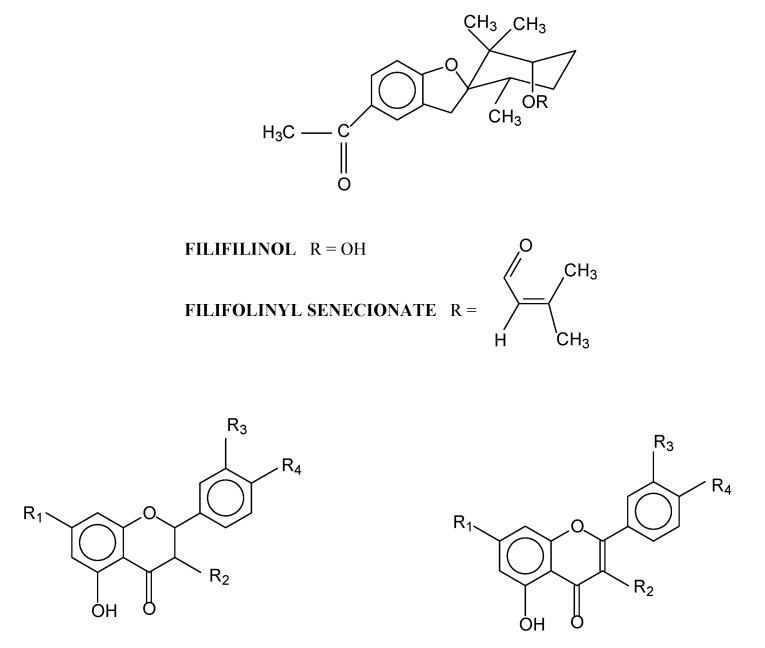
Structure of the compounds isolated from exudates resinous of *Heliotropium taltalense* Phil.

Like that of other species of *Heliotropium*, this resinous exudate was characterized by the presence of flavonoids and aromatic geranyl derivatives. From the resinous exudate of *H. taltalense* five compounds (Figure 1): two aromatic geranyl derivatives called filifolinol (**1**) and filifolinyl senecionate (**2**) were isolated. These had been previously obtained from *H. filifolium* (Miers) Reiche [2,6] and their antifungal and antiviral activities were demonstrated [22,23,24]. Also, three flavonoids – naringenin (**3**), 3-O-methylgalangin (**4**) and 7-O-methyleriodictiol (**5**) – were purified. These compounds were previously obtained from *H. sinuatum* (Miers) [3].

### 2.2. Antioxidant activity

The flavonoids (ethanolic solution) were added to a DPPH solution in homogeneous solvent (EtOH) and a micelar solution of SDS. The antioxidant activity of the flavonoids was interpreted in terms of the initial rate of the reaction. The results obtained (Table 1) using the microheterogeneous system follow the same tendency of the registered with ethanolic solution. The best ability to capture free radicals is displayed by 7-*O*-methyleriodictyol. This is attributed to the presence of a catechol system in its B ring, which makes it the best antioxidant due to a better radical stabilization caused by H-abstraction at position C-4’ forming a hydrogen bond with the phenolic hydroxyl group at C-3’ [17]. In second place came 3-*O*-methylgalangin, a compound with only two OH groups, and then naringenin with three. The higher antioxidant activity 3-*O*-methylgalangin can be attributed to the presence of C-2-C-3 unsaturation allowing the resonance stabilization of the formed radical, according to the analysis of spin density map obtained by Modak *et al*. [16].

**Table 1:** Initial rate for the reaction of DPPH radical with the flavonoids **3**-**5** in homogenous system (EtOH) and microheterogenous system (SDS).

Compounds	Concentration(mM)	V_i_ DPPH/EtOH (mM/s)	V_i_ DPPH/SDS (mM/s)
Naringenin	15.0	- 0.042220	- 0.06009
	30.0	- 0.028222	- 0.03791
	60.0	- 0.009550	- 0.01460
3-O-methylgalangin	3.00	- 0.001690	- 0.00245
	6.00	- 0.001790	- 0.00270
	12.00	- 0.002200	- 0.00302
7-O-methyleriodictyol	0.014	- 0.026210	- 0.03390
	0.028	- 0.038780	- 0.06061
	0.041	- 0.042500	- 0.10391

V_i_: initial rate

**Figure 2:**
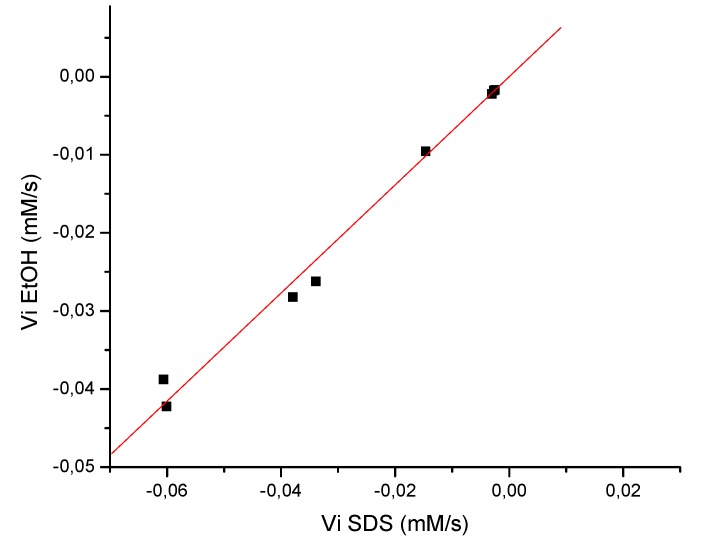
Graph of the initial velocity for the reaction of DPPH radical with the flavonoids **3**-**5** in homogenous system (EtOH) and microheterogenous system (SDS).

On the other hand for all flavonoids examined the initial rates (absolute values) were higher in SDS media than in ethanolic solution. In that respect, the literature proposes that this would be due to the fact that the surfactant molecule interacts with the flavonoid molecule, probably by H-bonding between the flavonoid and SDS molecule [18]. Liu [19] showed that in quercetin the most acidic phenolic OH group at C-7 in the A ring can dissociate to form quercetin anion, which has electrostatic repulsion with the negatively charged polar groups of SDS micelles, but there also exists hydrophobic interaction between SDS micelle and quercetin. In other work [18] the micelar phase was less polar than the aqueous phase and therefore the less hydrophilic flavonoids were more soluble than more hydrophilic ones. Based in these antecedents and the fact that 7-O-methyleryodictyol has two OH groups at C-3’ and C-4’ position that are involved in intra-hydrogen bonding, is less hydrophilic than naringenin and 3-O-methylgalangin, and consequently the flavonoid molecule penetrates very deeply in the SDS micelles, facilitating their interaction with the liposoluble radical DPPH. This argument allows also to explain why 3-O-methylgalangin improves its antioxidant activity using SDS at a higher level compared with naringenin. The first compound has two OH and one OCH_3_ groups while naringenin has three hydroxyl groups, increasing its hydrophilic character. The results of Table 1 also show that the flavonoids act in a different way, since it is observed that there is not a directly proportional relationship between the concentration and the rate. However, graph of initial rate in EtOH *vs*. initial rate in SDS (Figure 2) shows a straight line with a slope of 0.69272. This value indicates that the reaction in micelar phase is faster than the reaction in ethanolic solution.

Vi: initial rate; ■: initial rate values obtained from reaction in ethanolic solution and micelar phase of the compounds **3**-**5**.

In relation to the resinous exudate, three ethanolic solutions of different concentration were prepared. An aliquot of these solutions was added to a DPPH solution in a homogeneous solvent (EtOH) and to a micelar solution of SDS. Changes in the absorbance of the solutions containing the resinous exudate were measured at 517 nm. The antioxidant activity was expressed in terms of initial rate (Table 2). The resinous exudate improved the antioxidant activity in SDS as with the flavonoids. However, the resinous exudate isolated from *H. taltalense* is formed by a mixture of compounds of different structures and properties, such as flavonoids and aromatic geranyl derivatives, therefore is only possible to predict that the antioxidant activity is due to the presence of phenols (flavonoids) but is not possible to suggest any mechanism. Although, the presences of the nonpolar and liposoluble compounds in the resin probably help to improve the location in the micelle, since according to the literature, the micelar phase is less polar than the aqueous phase [18].

**Table 2:** Initial rate for the reaction of DPPH radical with the resin of *Heliotropium taltalense* in homogenous system (EtOH) and microheterogenous system (SDS).

Concentration of resin	Vi EtOH (mM/s)	Vi SDS (mM/s)
100 mg/mL	-0.012398	-0.114356
10 mg/mL	-0.011796	-0.035083
2 mg/mL	-0.006048	-0.009052

*Vi: initial rate*

## 3. Experimental

### 3.1. General

IR spectra were obtained on a Perkin Elmer 735-B spectrophotometer. ^1^H- (400 MHz), ^13^C- (100 MHz) and 2D NMR spectra were recorded in CDCl_3_ on a Bruker Avance DRX400 spectrometer with TMS as internal standard. UV absorbance was measured employing a Shimadzu UV-160 spectrophotometer. 

### 3.2. Plant material

*Heliotropium taltalense* (Phil.) Johnst was collected in September 2006 in Quebrada Matancilla (latitude 25º6’, longitude 70º27’) at a height of 400 m, Region of Antofagasta, Chile. A voucher specimen was deposited in Herbarium of the Faculty of Forest Sciences of the University of Chile (2076).

### 3.3. Chemicals

Standards of filifolinol, filifolinyl senecionate, naringenin, 3-*O*-methylgalangin and 7-*O*-methyl-eriodictiol were obtained from Laboratory of Natural Products Chemistry of the Faculty of Chemistry and Biology of the University of Santiago of Chile. 1,1-Diphenyl-2-picrylhydrazyl (DPPH) and sodium dodecyl sulfate (SDS) were purchased from Sigma–Aldrich Chemical Co (St. Louis, MO, USA). Dichloromethane, chloroform, ethanol, methanol, silica gel 60 (70–230 mesh ASTM; 63–200 lm) for CC and GF_254_ for analytical TLC were obtained from Merck Ltd. (Darmstadt, Germany). All reagents were of analytical reagent grade. 

### 3.4. Isolation and characterization of constituent of the resin

The fresh plant (1.1 kg) was dipped into dichloromethane for 30 s. The organic extract was concentrated in a rotatory evaporator to give a residue of 132.1 g of resin (12% based on the fresh plant). The resinous exudate was separated into 10 fractions by CC (silica gel, mixtures of increasing polarity of chloroform-methanol as eluents). Five known pure compounds were obtained by preparative chromatography from some of these fractions. Their structures were established by comparison of their spectroscopic data (^1^H- and ^13^C-NMR and UV) with those in the literature and by co-chromatography with authentic samples. The structures are showed in Figure 1. The compounds filifolinol (**1**, 33.0 mg) and filifolinyl senecionate (**2**, 28.0 mg) were obtained previously from *Heliotropium filifolium* (Miers) Reiche [2,6]. The flavonoids naringenin (**3**, 17.0 mg) 3-*O*-methyl-galangin (**4**, 88.0 mg) and 7-*O-*methyleriodictiol (**5**, 57.0 mg) were previously obtained from *Heliotropium sinuatum* (Miers) [3]. 

### 3.5. Antioxidant activity determination

The antioxidant activity of the flavonoids was determined evaluating the changes in the absorbance of an ethanolic solution of DPPH (75 μM) in response to different concentrations of the flavonoids, based on the method of Brand-Williams [25]. UV absorbance of the solutions was measured at 517 nm for 10 min at 25ºC. All determinations were performed in triplicate. In order to determine the effect of the concentration in the activity, three different concentrations for each compound were used. The rate of the reaction was followed by registering the absorbance of DPPH at 517 nm as a function of time. The estimation of the initial rate was obtained by fitting the absorbance decay, measured at 517 nm, to a uni-exponential function using the Origin^®^8 computer program [20]. Finally the first order derivative was calculated at time zero (time to which the first determination of absorbance is realized immediately after the compound was added). This value corresponds at the initial rate of the reaction. The results are shown in Table 1.

To determine the antioxidant activity using micelar media, the changes in the absorbance of a solution formed with SDS (50 mM, 3.0 mL) in phosphate buffer 20 mM, pH 7, and an aliquot of DPPH in ethanol to obtain a concentration 75 μM, were evaluated. Then, different concentrations of flavonoids were added to the solution DPPH/SDS and the activity was evaluated in the same way described previously. The results are shown in Table 1. The correlation of the initial rate in ethanolic solution and solution micelar is shown in Figure 2. The determination of the antioxidant activity with the resin was evaluated at three different concentrations (100, 10, 2 mg/mL) in the same way described previously. The results are shown in Table 2.

### 3.6. Statistical analyses

Statistical tests were performed using the Origin^®^8 computer program. Two sample hypothesis tests were carried out. Differences between pairs of means were assessed. Null hypothesis= velocity in EtOH-velocity in SDS ≥ 0. Alternative hypothesis = velocity in EtOH-velocity in SDS < 0. At the 0.05 level, the program determined that the difference of the population means was significantly less than the test difference (0). Therefore, the initial velocity obtained using DPPH in EtOH or DPPH in SDS for each concentration are statistically different.

## 4. Conclusions

Chemical analysis of exudates resinous isolated from *Heliotropium taltalense* Phil. was carried out for the first time. From the resin five compounds were identified: filifolinol and its ester derivative filifolinyl senecionate and the flavonoids naringenin, 3-*O*-methylgalangin and 7-*O*-methyleriodictiol. These types of compounds are characteristic of the resinous species that grow in a desert environmental.

The results obtained from the evaluation of the antioxidant activity of the flavonoids and resin in homogenous system (ethanolic solution) and microheterogeneous system (SDS micelles) allow us to conclude that the antioxidant molecules react more quickly when are inserted in the micelle, improving the activity. Besides with these results it is possible to propose that both hydrophobic effects and electrostatic interaction should be considered in relation to the solubilization of organic solutes as flavonoids in the ionic micelles. However, the impact of the hydrophobic effect for the flavonoids employed in this study on the partition would play a minor role on moderated hydrophobic compounds in micelles. These data and the discussion suggest that antioxidant capacity depends on environment and that results evaluated in homogeneous solution may not be extrapolated to a heterogeneous system.

On the other hand, although the values of antioxidant activity evaluated showed that the flavonoids in the resinous exudate were not highly reactive it is probable that the plant produces them at a high concentration, as a protection method against environmental aggressions.
